# Development of Lymphoproliferative Diseases by Hypoxia Inducible Factor-1alpha Is Associated with Prolonged Lymphocyte Survival

**DOI:** 10.1371/journal.pone.0057833

**Published:** 2013-04-12

**Authors:** Eisaburo Sueoka, Naoko Sueoka-Aragane, Akemi Sato, Masaru Ide, Hideaki Nakamura, Yusuke Sotomaru, Choji Taya, Hiromichi Yonekawa, Tomoyuki Kitagawa, Yasushi Kubota, Shinya Kimura, Kei Nakachi, Keiji Tanimoto

**Affiliations:** 1 Department of Internal Medicine, Faculty of Medicine, Saga University, Saga, Japan; 2 Department of Radiation Biology, Research Institute for Radiation Biology and Medicine, Hiroshima University, Hiroshima, Japan; 3 Natural Science Center for Basic Research and Development, Hiroshima University, Hiroshima, Japan; 4 The Tokyo Metropolitan Institute of Medical Science, Tokyo, Japan; 5 Cancer Institute Hospital, Tokyo, Japan; 6 Radiation Effects Research Foundation, Hiroshima, Japan; Baylor college of Medicine, United States of America

## Abstract

Hypoxia-inducible factor-1alpha (HIF-1 alpha) plays an essential role in the regulation of various genes associated with low oxygen consumption. Elevated expression of HIF-1alpha has been reported to be associated with tumor progression, invasion and metastasis in many cancers. To investigate the role of HIF-1alpha in tumor development and metastasis, we established transgenic mice constitutively expressing *HIF1A* gene under regulation of the cytomegalovirus gene promoter. Although HIF-1alpha protein levels varied among organs, expression of *HIF1A* mRNA in most organs gradually increased in an age-dependent manner. The transgenic mice showed no gross morphological abnormality up to 8 weeks after birth, although they subsequently developed tumors in the lymphoid, lung, and breast; the most prominent tumor was lymphoma appearing in the intestinal mucosa and intra-mesenchymal tissues. The prevalence of tumors reached 80% in 13 months after birth. The constitution of lymphocyte populations in the transgenic mice did not differ from that in wild-type mice. However, lymphocytes of the transgenic mice revealed prolonged survival under long-term culture conditions and revealed increased resistance to cytotoxic etoposide. These results suggest that HIF-1alpha itself is not oncogenic but it may play an important role in lymphomagenesis mediated through the prolonged survival of lymphocytes in this transgenic mouse model.

## Introduction

Hypoxia, is a common feature of various cancers [1]. Cells under hypoxic conditions develop numerous adaptive responses to hypoxic stress concurrently with altered expression of hundreds of genes that are regulated by hypoxia inducible factors (HIFs) [1],[2]. HIF-1alpha is a transcription factor forming a heterodimer with a constitutively expressed HIF-1beta subunit. The structurally- and functionally-related HIF-2alpha protein also binds to HIF-1beta. These heterodimers regulate target genes by binding to a consensus sequence called hypoxia responsive element (HRE) [1]–[3]. Under nonhypoxic conditions, HIF-1alpha is modulated by O_2_-dependent prolyl hydroxylase (PHD) and recognized by von Hippel-Lindau (VHL) tumor suppressor protein, resulting in recruitment of a ubiquitin ligase complex and subsequent proteasomal degradation of HIF-1alpha [2]–[4]. However, under hypoxic conditions, reduced hydroxylation activity causes a decrease in ubiquitination, leading to accumulation of HIF-1alpha [3]–[5]. Immunohistochemical analyses of various tumor specimens have demonstrated increased amounts of HIF-1alpha protein in tumor cells surrounding the necrotic tissues in hypoxic areas [6]–[10].

In addition to hypoxic conditions in tumor tissues, selected genetic alterations in cancer cells also enhance HIF-1 activity, typically mutations in, or loss of, *VHL* gene in clear cell renal cell carcinoma [9],[11]. Activation of phosphatidylinositol-3-kinase (PI3K) pathway or inactivation of tumor suppressor genes such as p53 has been reported to enhance HIF-1 activity in cancer cells [12],[13]. Any these changes cause increased basal levels of HIF-1alpha in cancer cells, characterizing physiological response to hypoxia. Overexpression of HIF-1alpha is often associated with poor prognosis, inferring that HIF-1alpha plays an important role in various stages of cancer progression, including immortalization, maintenance of stem cell pools, genetic instability, neovascularization, invasion/metastasis, and resistance to treatment [14]–[22]. Regarding the role of HIFs in tumorigenesis, HIF-1alpha deficiency was associated with delayed tumor growth in subcutaneous xenograft models using immunocompromised mice. In addition, transformed *Hif1a* ^−/−^ mouse embryonic fibroblasts grew slower and formed less vascularized tumors than wild-type fibroblasts. These results suggest that HIF-1alpha acts on both tumor growth and angiogenesis [23].

The role of HIF-1alpha in carcinogenesis has not yet been clarified. Considering that expression of HIF-1alpha increases from the early stage of cancer [7],[14],[24], HIF-1alpha may play a role in the process of carcinogenesis. Bertout et al. recently demonstrated that heterozygous deletion of *HIF1A* gene reduced the occurrence of thymic lymphoma in p53 mutant mice [24]. They also reported that decreased HIF-1alpha levels were associated with impairment of Notch signaling, resulting in decreased induction of Notch target genes. A recent study by Liao et al. demonstrated that HIF-1alpha is not required for tumor initiation, but loss of HIF-1alpha caused tumor latency and decreased proliferation, angiogenesis, and metastatic potential using a mouse breast cancer model [25]. In addition, accumulating evidence suggests that HIFs are critical for maintaining the population of stem cell-like tumor cells (“cancer stem cells”) that are associated with recurrence, metastasis, and resistance to conventional treatments [17],[18]. The role of HIF-1alpha in tumorgenesis has thus far been investigated in limited phases of tumor development such as metastasis or angiogenesis. Further studies based on spontaneous tumor model are warranted to assess diversified role of HIF-1alpha in cancer development.

We therefore established a transgenic mouse model overexpressing *HIF1A* coding sequence under control of the cytomegalovirus (CMV) gene promoter, thereby broadening expression of HIF-1alpha in various tissues. This model allows us to examine the effect of overexpressed HIF-1alpha in individual tissues as well as mechanisms of adaptation to hypoxic conditions of tumors.

## Materials and Methods

### Animal model

**Figure 1:**
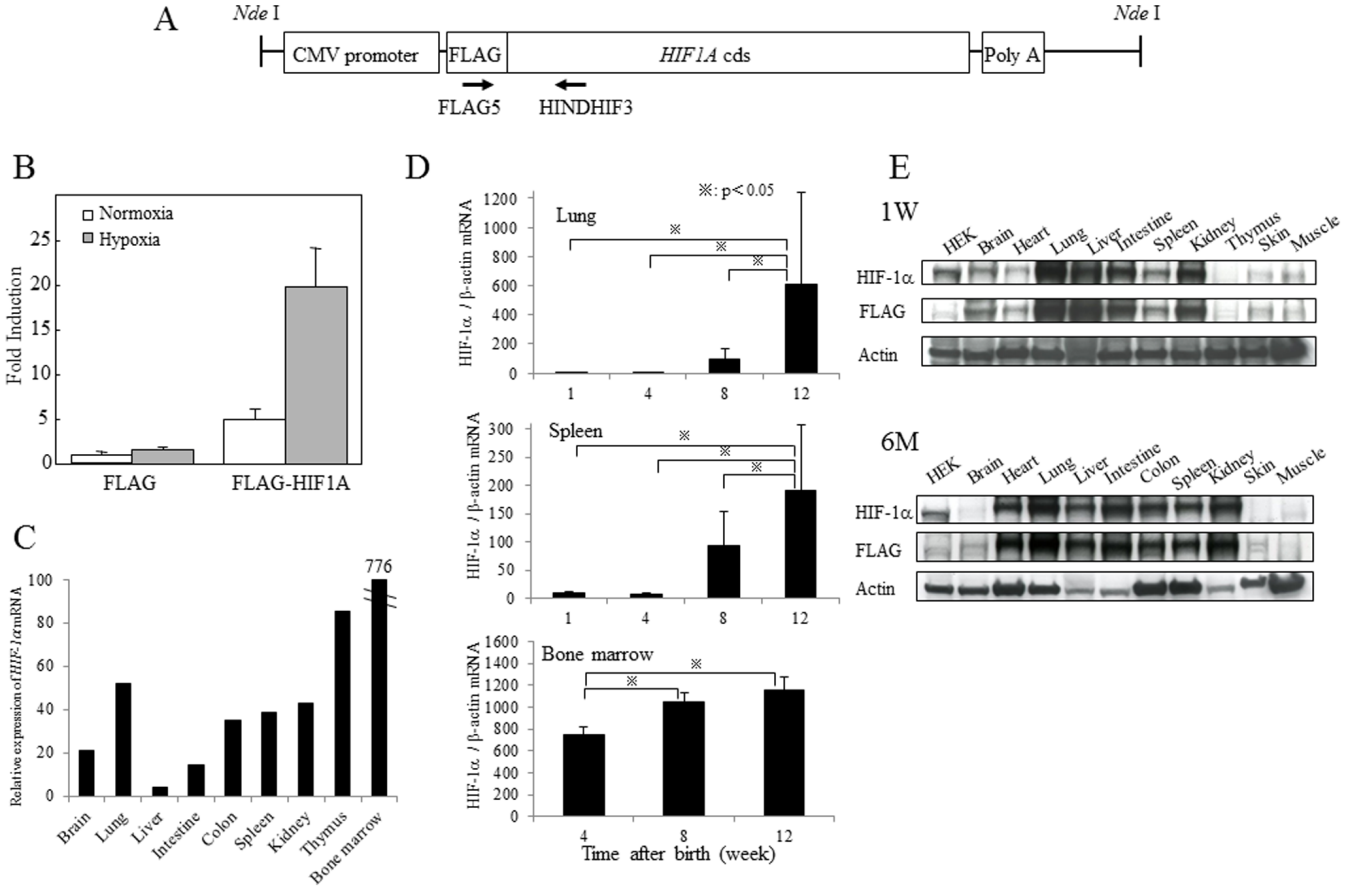
The construct of transgene and expression of human HIF-1alpha in the transgenic mouse tissues. (A) The construct of chimeric gene including human *HIF1A* cDNA fused with the *FLAG* gene at the 5′ end of the *HIF1A* gene. (B) Activation of human HIF-1alpha protein in murine cells was confirmed by luciferase activity in BALB/3T3 cells stably transfected with the transgenic construct. (C) Ectopic expression of human *HIF1A* mRNA was determined by real-time RT-PCR as described in Materials and Methods. (D) Sequential analyses of expression levels by real-time RT-PCR in various tissues of transgenic mice. Relative expression of human *HIF1A* mRNA was assessed by the level of *Actb* mRNA. (E) Expression of HIF-1alpha protein in various organs was determined by western blotting with use of both anti-HIF-1alpha and anti-FLAG antibodies.

Mice were kept under pathogen-free conditions in animal facilities at Saga University and Hiroshima University according to institutional guidelines. The protocol was approved by the Committee on the Ethics of Animal Experiments of the Saga University (Permit Number:15-005-1) and Hiroshima University (Permit Number: 22–125). All surgery was performed under sodium pentobarbital anesthesia, and all efforts were made to minimize suffering. The transgenic mouse carries a chimeric gene including a fragment from the promoter region of the CMV gene and all coding sequences of human *HIF1A* gene fused with the *FLAG* gene at the 5′ end of the *HIF1A* gene (*HIF1A* TG mice; Fig. 1A). Six strains of transgenic mice were obtained and backcrossed onto the BALB/c genetic background mice for more than 10 generations. To determine the presence of the transgene in the backcrossed mice, we performed PCR analysis of genomic DNA obtained from tails at the age of 4 weeks using a primer set specific to the FLAG tag: 5′-ATG GAC TAC AAA GAC GAT GAC GAC AAG-3′ (FLAG5), and another set specific to the human *HIF1A* gene: 5′-ATT CTG AGA AAA AAG CTT CGC TGT GTG-3′ (HINDHIF3) (Fig. 1A). Transgene copy number was estimated by real-time PCR, following which similarities of *HIF1A* mRNA expression level and phenotype were confirmed using one line of transgenic founder mice.

### Blood count

Peripheral blood was collected and analyzed on an automated blood cell counter, KX-21 (Sysmex), according to the manufacturer's instructions.

### Chemicals and antibodies

Lipopolysaccharide (LPS), 12-*O*-tetradecanoylphorbol 13-acetate (TPA), and ionomycin purchased from Sigma-Aldrich (Tokyo, Japan) were dissolved in distilled water (LPS) or dimethyl sulfoxide (TPA and ionomycine) before use. IgM was purchased from Wako chemical Co. (Tokyo, Japan). Sources of antibodies were as follows: HIF-1alpha from BD Biosciences, FLAG from SIGMA, and actin from Santa Cruz Biotechnology, Inc. for western blotting; CD45R from Santa Cruz Biotechnology, Inc. and CD3 from DAKO Co. for immunohistochemistry.

### Aging Study

Cohorts were produced by mating *HIF1A* TG mice and were compared to wild-type mice. Since the *HIF1A* TG mice were backcrossed to BALB/c background for more than 10 generations, all mice are presumed to have a similarly mixed background. Mice were evaluated daily for signs of morbidity or tumor growth. Distressed mice were euthanized with ether and dissected. All soft tissues were divided into 3 parts: two parts were frozen in liquid nitrogen for storage until evaluation by RT-PCR or western blotting and one part was fixed in 10% formaldehyde and processed for immunohistochemistry. Tumors were identified by veterinary pathologists.

### Cell preparation and culture conditions

Thymocytes, spleen cells, and lymphocytes in Peyer's patches were isolated from *HIF1A* TG mice and wild-type mice at the ages of 1, 4, 6, and 12 months. Analysis of growth capacity in lymphocytes from spleen, thymus, and Peyer's patches in the intestine proceeded as follows. Spleen cells were separated into T and B cells using negative selection by the MACS system. Splenic B cells were stimulated by LPS or IgM for 48 hours, and splenic T cells or thymocytes were stimulated by TPA plus ionomycin for the same hours. Spleen cells were further purified to T and B cell rich-fractions using the MACS® pan-T isolation kit and a B-cell isolation kit (Miltenyi Biotec K.K., Tokyo Japan). Cells were cultured in RPMI1640 medium containing 10% fetal bovine serum and antibiotics. For the cell proliferation assay, 5×10^4^ trypan-blue-negative lymphocytes were plated in 96-well culture plates containing RPMI1640 medium supplemented with 10% FBS in the presence or absence of mitogens such as LPS or IgM for B cells and TPA, ionomycin, or CD3 antibody for T cells. As short term culture, 48 h after seeding, cell proliferating activity was determined by Cell Proliferation ELISA, BrdU (chemiluminescence) kit (Roche Diagnostics K.K., Tokyo, Japan) according to the manufacturer's instructions. Relative amount of cell growth was calculated as a ratio of number of cells treated with mitogens to number of untreated cells, and is shown as mean ± SD of six wells for cells isolated from five mice.

*In vitro* colony-forming assays were performed in duplicate by plating 500 bone marrow cells with 1 ml of MethoCult™ M3434 medium (StemCell Technologies) in 35 mm Petri dishes. Colonies were counted after 4–5 days by May-Grünwald Giemsa staining.

### Fluorescence-activated cell sorter (FACS) analysis

Thymocytes, spleen cells, and lymphocytes from Peyer's patches obtained from 1- to 12-month-old mice were counted and stained with fluorochrome-conjugated monoclonal antibodies using standard procedures. Acquisition was performed with a FACSCalibur® (BD Biosciences) and results were analyzed by FlowJo soft-wear (Tomy Digital Biology Co., Ltd, Tokyo, Japan). Antibodies were anti-mouse against CD3e, CD4, CD8, CD19, B220, CD25, CD44, IgM (eBiosciences).

### Real-time RT-PCR and western blot analysis

Total RNA was extracted from each tissue using an RNeasy mini kit (QIAGEN) and 50–500 ng total RNA was reverse transcribed into cDNA using the High Capacity RNA-to-cDNA Master Mix (Applied Biosystems) following the manufacturer's instructions. Real-time PCR was performed on the Applied Biosystems StepOnePlus® Real-Time PCR System (Applied Biosystems) using the TaqMan® Gene Expression Assay (Applied Biosystems). PCR reaction proceeded at fast mode: 95°C for 20 sec followed by 40 cycles at 95°C for 1 sec and 60°C for 20 sec. Primer sets were as follows: human *HIF1A* (Hs00153153), and *Actb* (Mm00607939) (TaqMan® Gene Expression Assays, Inventoried). For the cDNA microarray analysis, 0.5 μg of total RNA was extracted from T and B lymphocytes obtained from mouse spleen, and they were analyzed using 3D-GeneTM Mouse Oligo chip 24k labeled with 2-color hybridization system (Toray Industries, Inc., Tokyo, Japan).

Western blotting was performed using whole-cell lysates prepared from lymphocytes or mouse tissues using lysis buffer containing 50 mM Tris-HCL at pH 8.0, 150 mM NaCl, 5 mM MgCl_2_, 1% TritonX-100, 0.1% sodium dodecyl sulfate, 0.5% sodium deoxycholate, 40 mM sodium fluoride, 1 mM sodium orthovanadate, 1 µg/ml leupeptin, 10 µg/ml aprotinin, and 1 mM phenolmethylsulfonyl fluoride, as reported previously [26]. Protein was separated using a 10% NuPAGE electrophoresis system (NOVEX, San Diego, CA), transferred to a nitrocellulose membrane (Schleicher & Schuell, Inc., Keene, NH), blocked with 5% milk at 4°C overnight, and finally reacted with primary antibodies. An ECL kit (Amersham Corp., Arlington Heights, IL) was used for detection.

## Results

### Generation of *HIF1A* transgenic mice and expression of *HIF1A* transgene in each tissue

Levels of HIF-1alpha protein are regulated with a balance between their protein syntheses and degradations. Although it is not fully activated, excessive expression of wild-type *HIF1A* gene is enough to work in normoxic conditions as we previously reported [27], [28]. In fact, activation of human HIF-1alpha protein, which was used for generation of the transgenic mice, in murine cells was confirmed by luciferase activity in BALB/3T3 cells in both normoxic and hypoxic conditions (Fig. 1B). We, therefore, forwarded generationsof *HIF1A* transgenic mice and established six strains from littermates of transgenic founder mice (BALB/c) and backcrossed to BALB/c mice at least 10 generations. All of those strains developed normally, and the transgene was passed to offspring following a Mendelian inheritance. Since copy number and expression level of *HIF1A* gene are not mutually distinguishable among strains, we used one strain for further analyses. We first determined the expression of *HIF1A* gene and protein in various tissues by concurrent use of real-time RT-PCR and western blotting. Ectopic expression of human *HIF1A* mRNA was observed in various organs (Fig. 1C). Expression was detected from 1 week after birth and gradually increased from 8 weeks over time by mRNA levels (Fig. 1D). Unexpectedly, expression levels of HIF-1alpha protein varied among organs (Fig. 1E), although the *HIF1A* gene regulated by the CMV promoter was overexpressed in all organs we examined. High levels of expression of HIF-1alpha protein were observed in the heart, lung, spleen, kidney, and skin at the age of 6 months, in terms of western blotting with use of both anti-HIF-1alpha and anti-FLAG antibodies.

### Characteristics of phenotypes in hematopoietic and lymphoid systems

Noting that high levels of expression of *HIF1A* mRNA were observed in the bone marrow of *HIF1A* TG mice, we analyzed induction of erythropoietin, a target gene of HIF-1alpha and peripheral-blood erythrocyte count. Colony forming activity for CFU-E was slightly higher in *HIF1A* TG mice. However, concentrations of serum erythropoietin were not elevated, and erythrocyte count did not differ from that in wild-type mice (Fig. S1).

### Tumor development in transgenic mice

**Figure 2:**
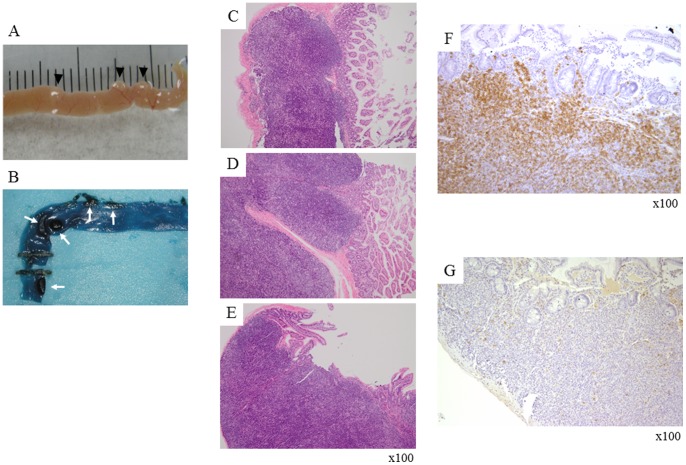
Lymphoproliferative diseases in *HIF1A* TG mice. (A) Macroscopic appearance of intestinal tumors. (B) The number of enlarged Peyer's patches per mouse was determined by Methylene Blue staining. (C–E) Some enlarged abdominal lymph nodes were found with extravasation from the capsules. Immunohistochemical analyses in parallel showed that CD45R-positive (F), and CD3- negative (G) B-cells predominated in the enlarged Peyer's patches.

**Figure 3:**
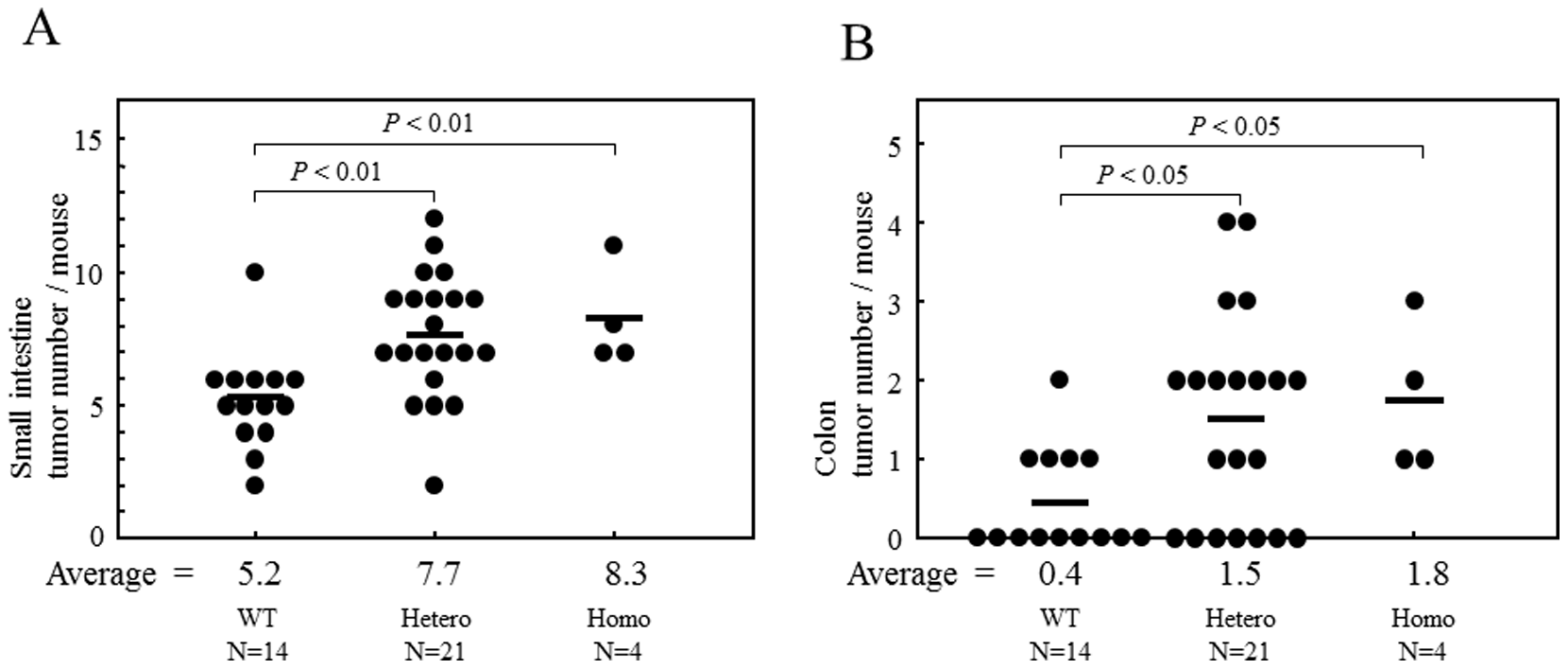
The number of intestinal tumors in *HIF1A* TG mice. The number of enlarged Peyer's patches per mouse (size greater than 3 mm, determined by Methylene Blue staining) increased in both the small intestine (A) and colon (B) of *HIF1A* TG mice, compared with that found in wild-type or BALB/c mice (p<0.01 and 0.05, respectively).

*HIF1A* TG mice were born without phenotypic abnormalities and grew normally, although some of the mice showed weight loss in four months after birth and died in a cachexic state. Autopsy revealed that these dead mice often suffered from tumors such as numerous abdominal nodules on the surfaces of the small intestine and colon, and lymph node enlargement, and lung tumors. Consecutive macroscopic examination at autopsy clarified that abdominal nodules were enlarged Peyer's patches spreading throughout the intestinal tract (Fig. 2A–G). Further investigation focusing on lymphoid involvement revealed that the number of enlarged Peyer's patches per mouse (size greater than 3 mm, determined by Methylene Blue staining) increased in both the small intestine and colon of *HIF1A* TG mice, compared with that found in wild-type or BALB/c mice (p<0.01 and 0.05, respectively) (Fig. 3A, B). Furthermore, homozygous *HIF1A* TG mice evidenced a larger number of enlarged Peyer's patches than heterozygous or wild-type mice. Immunohistochemical analyses in parallel showed that CD45R-positive B-cells predominated in the enlarged Peyer's patches (Fig. 2F, G).

**Figure 4:**
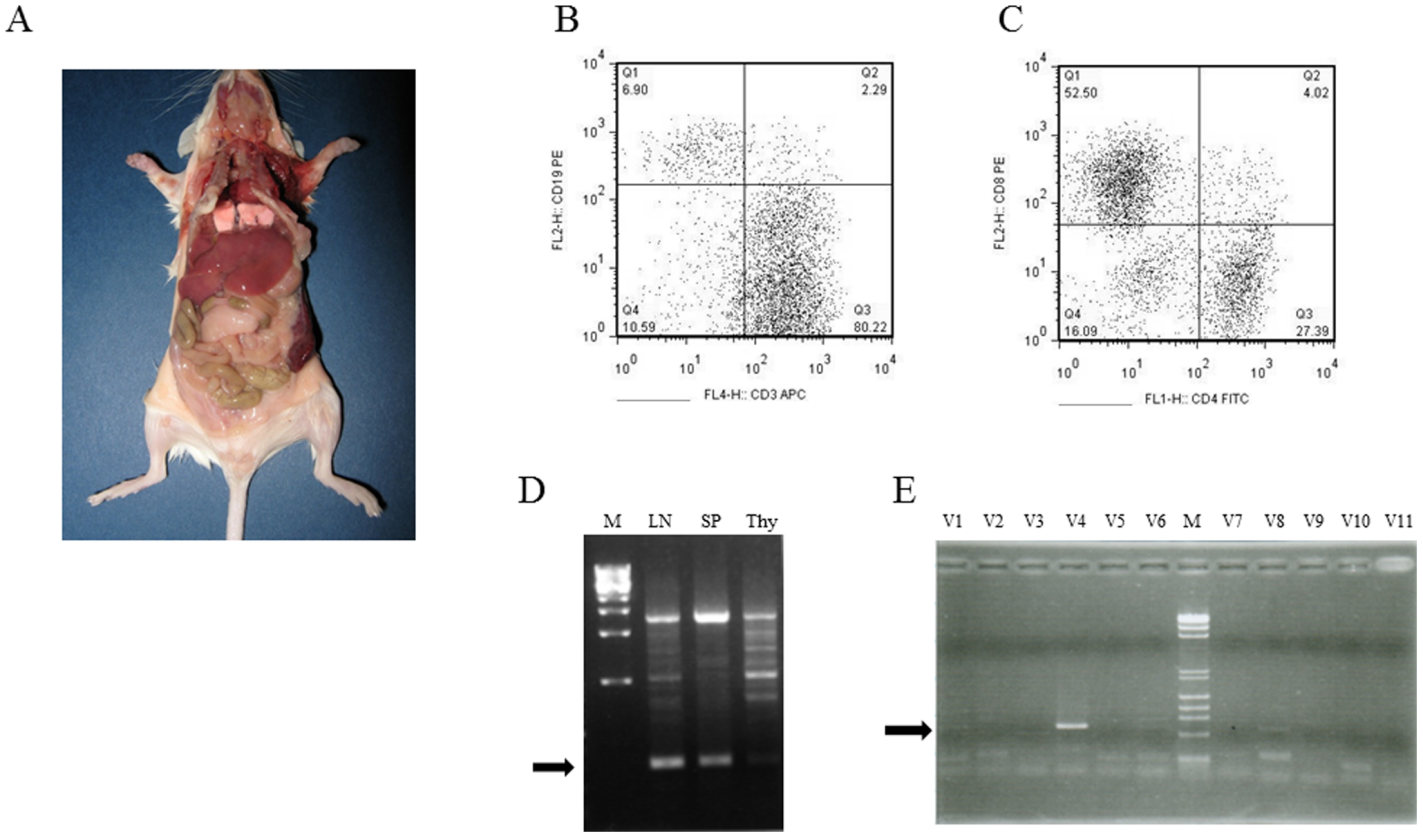
Monoclonal rearrangement of T cell receptor gene in a tumor in a *HIF1A* TG mouse. (A) A mouse showing enlarged peritoneal lymph nodes and hepatosplenomegaly. Monoclonality of proliferating lymphocytes was analyzed by flow cytometry with double staining using CD3 and CD19 (B), or CD4 and CD8 (C). (D) Monoclonal rearrangement of TCR gene in lymphocytes from a peritoneal lymph node and spleen. (E) PCR using primer sets specific for V, D, and J regions of T cell receptor (*TCR*) gene.

Lymphoproliferative disorders were sorted into benign lymphopherative diseases and lymphoma, and their incidence was compared between *HIF1A* TG mice (homozygotes and heterozygotes) and wild-type mice in Table 1. Here, lymphoproliferative diseases include Peyer's patches with size greater than 3 mm, enlargement of lymph nodes, and infiltration of lymphocytes into organs; lymphoma was diagnosed when enlarged abdominal lymph nodes were found with extravasation from the capsules and accompanied with systemic infiltration of lymphocytes into organs such as lung and liver. Lymphoproliferative diseases and lymphoma were found in 81% and 44% of *HIF1A* TG homozygous mice, 81% and 38% of the heterozygotes, and 17% and 8% of wild-type mice, respectively (p<0.01 for heterozygotes vs. WT on lymphoproliferative diseases). Monoclonality of proliferating lymphocytes was analyzed by PCR using primer sets specific for immunoglobulin heavy chain (*IgH*) gene and V, D, and J regions of T cell receptor (*TCR*) gene, or by flow cytometry. Among mice showing systemic infiltration of lymphocytes, most tumors evidenced a polyclonal pattern by PCR or flow cytometry. However, one *HIF1A* TG mouse showed proliferation of CD3-positive T lymphocytes (CD-4 positive cells predominated, Fig. 4A–C), with a monoclonal pattern of gene rearrangement in *TCR* (Fig. 4D, E), suggesting that the tumor cells were of monoclonal origin from alpha/beta type T cells.

**Table 1:** Incidence of lymphoproliferative disorders in transgenic mice.

TG mice	Lymphoproliferative disease	Lymphoma (B, T, non B/T)<$>\raster(80%)="rg1"<$>
Homo (n = 16)	12/16 (81%)	7/16 (44%)
		(5, 1, 1)
Hetero (n = 21)	17/21 (81%)	8/21 (38%)
		(5, 1, 2)
Wild (n = 12)	2/12 (17%)	1/12 (8%)
		(1, 0, 0)

^<$>\raster(80%)="rg1"<$> ^Monoclonality assessed by IgH or TCR rearrangement and/or systemic involvement of lymphoid cells was diagnosed as lymphoma.

**Figure 5:**
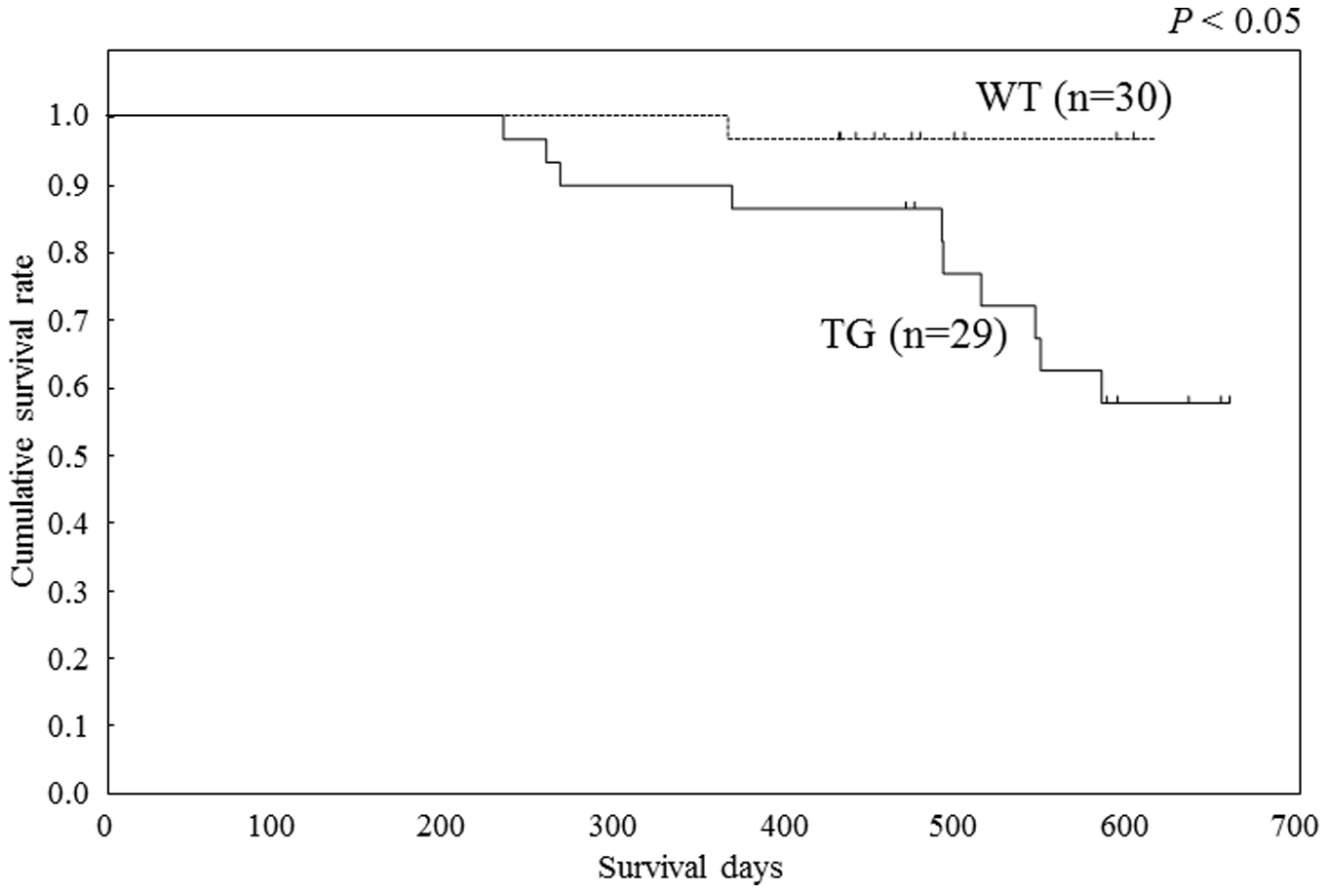
Overall survival of *HIF1A* TG mice. Overall survival of *HIF1A* TG heterozygous mice was shorter than that of wild-type mice in 24-month follow-up (p<0.05).

Overall survival was studied by comparing the *HIF1A* TG heterozygous and wild-type mice. Overall survival of *HIF1A* TG heterozygous mice was shorter than that of wild-type mice in 24-month follow-up (p<0.05) (Fig. 5). These results indicate that overexpression of HIF-1alpha is associated with tumorigenesis, specifically increased incidence of lymphoproliferative diseases and lymphoma development.

### Proliferating and survival potential of lymphocytes

**Figure 6:**
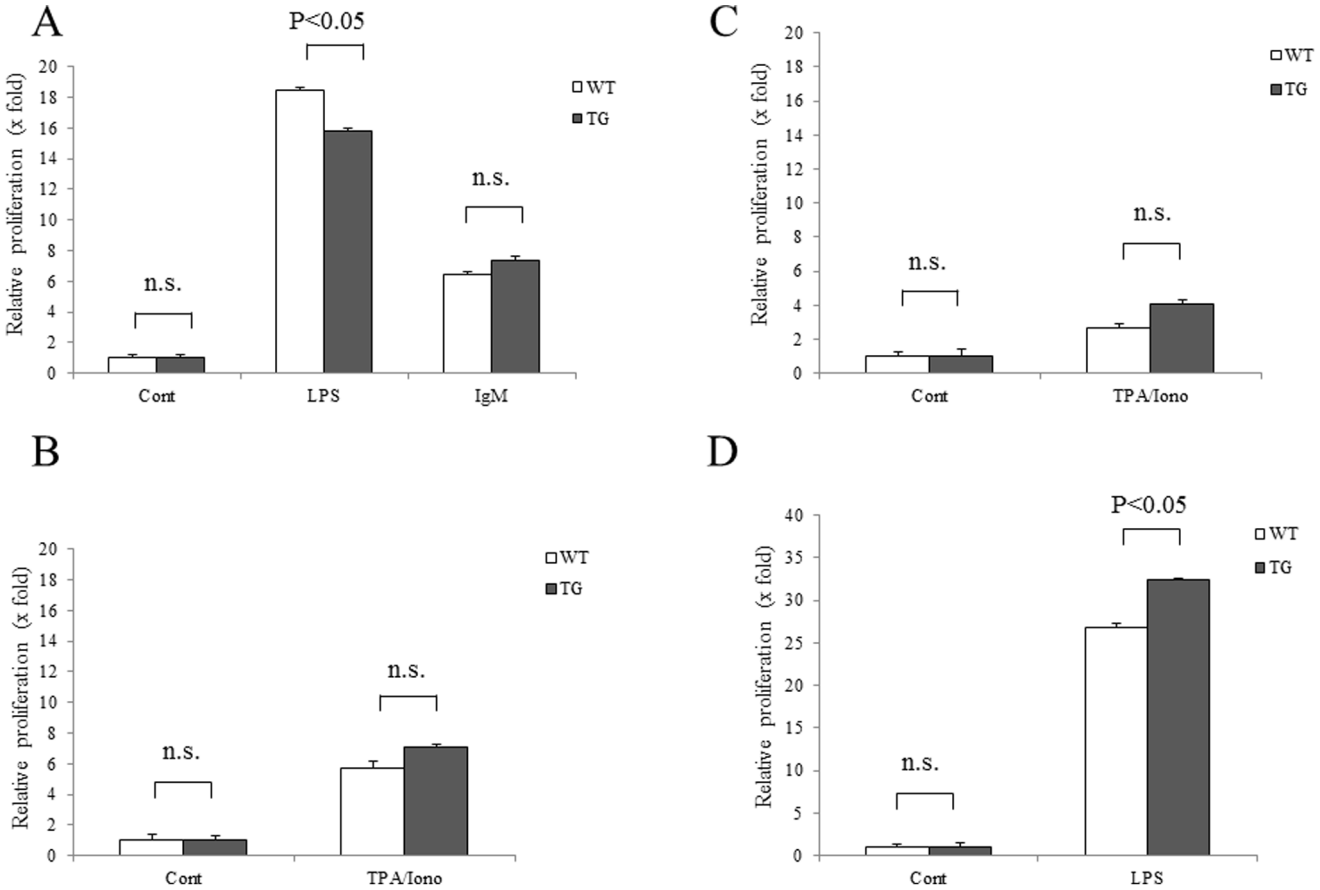
Proliferating and survival potential of lymphocytes from *HIF1A* TG mice. Proliferation rates were determined for splenic B cells from *HIF1A* TG and wild-type mice in terms of BrdU incorporation indices, although no clear differences were found between them. Proliferation rates of splenic B cells stimulated by LPS or IgM (A), splenic T cells stimulated by TPA and ionomycine (B), thymocytes stimulated by TPA and ionomycine (C), or B cells from Peyer's patchs stimulated by LPS (D).

We next examined the phenotype and proliferative capacity of lymphocytes from the *HIF1A* TG mice, since lymphoproliferative diseases and lymphomas were frequently observed in the mice. Phenotypic features of the lymphoid system were analyzed, in terms of T and B cell populations and subpopulations of T cells in the spleen as well as maturation pattern of thymic T cells, although gross abnormality was not observed (Fig. S2A–C). Proliferation rates were determined for splenic B cells from *HIF1A* TG and wild-type mice in terms of BrdU incorporation indices, although no clear differences were found between them. However, when the cells were stimulated with LPS and cultured for 48 hours, growth rates revealed significant difference: LPS-stimulated splenic B cells of the TG mice grew more slowly than those of wild-type mice (p<0.005) (Fig. 6A). In contrast, both splenic and thymic T cells of the TG mice showed slightly faster growth than those of wild-type mice after stimulation with TPA in combination with ionomycine (p<0.005) (Fig. 6B, C). In addition, B cells from Peyer's patches demonstrated faster growth in the TG mice than those in wild-type mice (p<0.005) (Fig. 6D).

**Figure 7:**
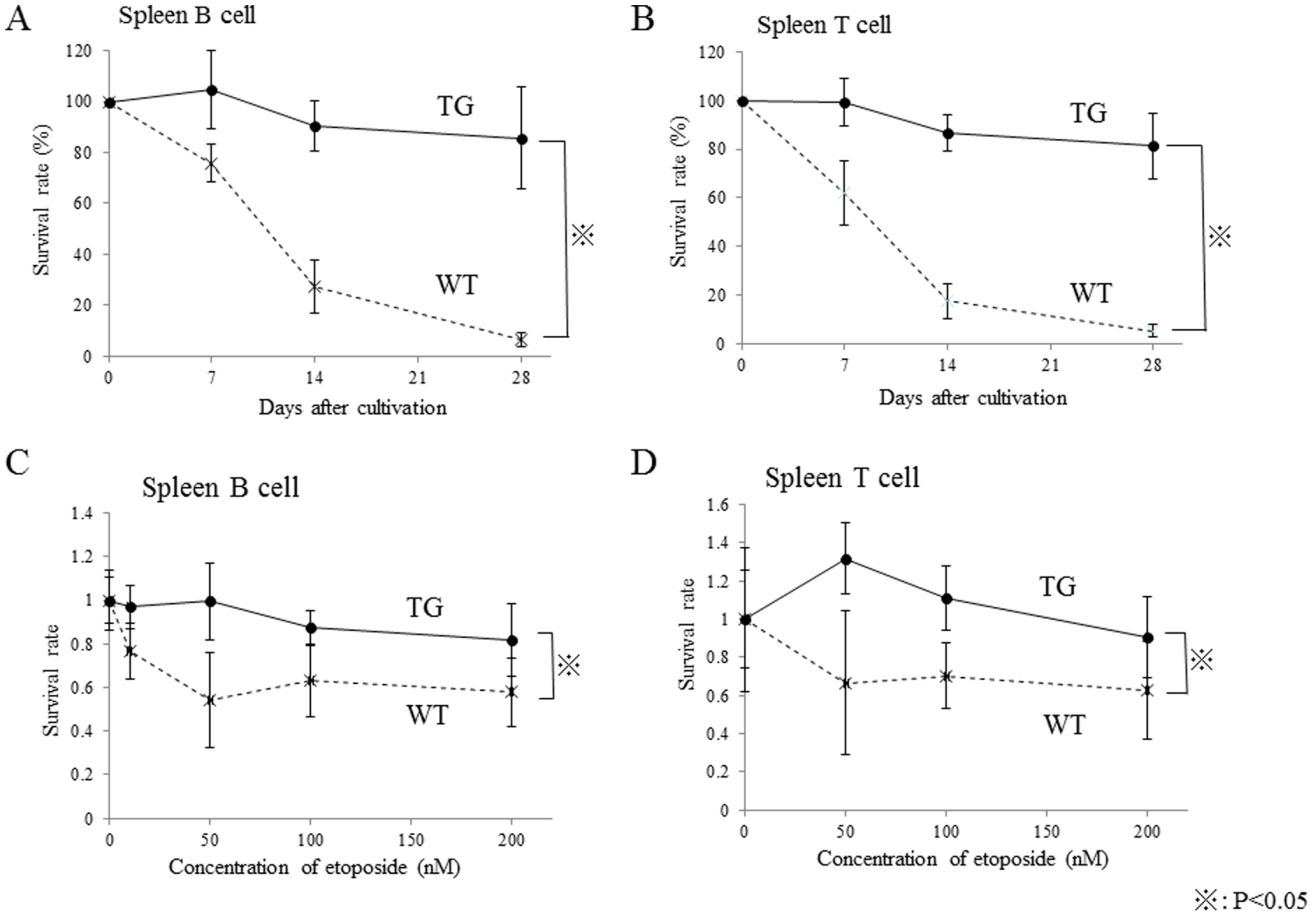
Prolonged survival of lymphocytes from *HIF1A* TG mice. Splenic lymphocytes were cultured under non-stimulating conditions for 28 days. Survival rates of splenic B cells (A) and splenic T cells (B). The declining slope was remarkably lower in *HIF1A* TG mice than in wild-type mice. (C, D) The sensitivity of *HIF1A* TG mice lymphocytes to etoposide, a topoisomerase II inhibitor.

These cells were also cultured under non-stimulating conditions for 28 days. Although the number of lymphocytes from both *HIF1A* TG mice and wild-type mice gradually decreased with days after cultivation, the declining slope was remarkably lower in *HIF1A* TG mice than in wild-type mice (Fig. 7A, B). These results suggest that HIF-1alpha overexpression prolongs lymphocyte survival.

Cells overexpressing HIF-1alpha have been thought to be resistant to genotoxic stresses such as chemotherapeutic agents and radiation. Finally, we studied the sensitivity of *HIF1A* TG mice lymphocytes to etoposide, a topoisomerase II inhibitor. It was found that *HIF1A* TG mice lymphocytes were more resistant to etoposide than wild-type mice lymphocytes (Fig. 7C, D).

## Discussion

To investigate the role of HIF-1alpha in spontaneous tumorigenesis, we established a *HIF1A* TG mouse model which constitutively and systemically overexpressed human HIF-1alpha. We found that *HIF1A* TG mice developed significantly-increased number of lymphoproliferative diseases, which were characterized by aggressive phenotypes such as involvement of multiple organs, invasion into adjacent tissues, and peripheral blood infiltration.

Expression levels of human HIF-1alpha varied among organs despite regulation by the CMV promoter. One possible mechanism is that protein degradation of HIF-1alpha differed among organs. However, levels of PHD and VHL proteins were substantially unchanged among organs, although expressions of other components of VHL ubiquitin ligase complex were not determined (data not shown). Although *HIF1A* mRNA was expressed at different levels among organs, its levels gradually increased in a time-dependent manner after birth. These data suggest that the amount of HIF-1alpha protein in *HIF1A* TG mice was regulated by both transcriptional and post-transcriptional modifications. Surprisingly, *HIF1A* TG mice did not show clear differences in serum erythropoietin concentration or peripheral-blood red cell counts, despite of *in vitro* experiments demonstrating that human HIF-1alpha expression could activate HRE containing promoter in a mouse cell line. A further investigation is needed to explain these findings.

The main phenotypical abnormality observed in *HIF1A* TG mice was development of lymphoproliferative diseases, which appeared from 3–4 months after birth. The majority of these tumors were located in the intestine, but some tumors displayed involvement in multiple organs accompanied with peripheral blood infiltration. Histological findings indicated that the proliferating lymphocytes evidenced a monotonous phenotype by immunohistochemistry and invaded from the lymph node capsules into the adjacent tissues. We defined such tumors as lymphoma and further investigated the rearrangement patterns of *TCR* and *IgH* genes. Monoclonal rearrangements of tumor-related genes in these tumors were rare, although one tumor showed a highly aggressive phenotype and was found to have a single rearrangement band for *TCR* gene (Fig. 4D, E). These results suggest that constitutive expression of HIF-1alpha promotes the occurrence of lymphoproliferative diseases, resulting in progression to overt lymphoma. Although the precise role of HIF-1alpha in lymphomagenesis is not clear at present, it is inferred that HIF-1alpha acts as a tumor promoter because the occurrence of lymphoma is having long latency events. Some recent reports suggest that HIF-2alpha also acts as a tumor promoter, in both *in vitro* and *in vivo* (*Tp53H/H* mouse) models [29]–[31]. In our mouse model, HIF-2alpha level was not altered by HIF-1alpha overexpression, indicating either that HIF-2alpha is not important for spontaneous tumorigenesis in the *Tp53H/H* mouse model, or that HIF-2alpha overexpression promotes tumor formation only in susceptible tissues.

In regards to the mechanisms of lymphomagenesis promoted by HIF-1alpha, we investigated several phenotypic features of lymphocytes from the *HIF1A* TG mice. First, abnormality in phenotypical development of T and B cells was not detected in the thymus, spleen, or bone marrow of the TG mice. Second, cell proliferation capacity differed between *HIF1A* TG and wild type. Thymocytes and splenic T cells of the TG mice evidenced enhanced mitogen-stimulated growth in short term (48 hr) culture, whereas the growth response of splenic B cells induced by LPS or IgM did not differ substantially. A more prominent difference between *HIF1A* TG and wild type mice was observed in long term (28 days) culture. The number of viable lymphocytes from wild-type mice rapidly decreased with days after cultivation, while B and T cells of the TG mice showed low declining slopes – remarkably prolonged cell survival (Fig. 7A, B). Furthermore, lymphocytes of the TG mice were resistant to cytotoxic etoposide (Fig. 7C, D). These results suggest that overexpression of HIF-1alpha remarkably affects lymphocyte survival in *in vitro* culture under normoxic conditions, although it has only a marginal effect on lymphocyte cell growth. Given the potential implications of our findings, a further analysis is warranted to identify such genes that are induced by constitutive activation of HIF-1alpha and responsible for prolonged survival of lymphocytes and consequently the occurrence of lymphoma. In fact, our preliminary gene expression profiling experiments found enhanced expression of selected anti-apoptotic genes in both splenic T and B cells of the TG mice (Table S1). We have not obtained clear results of alteration of *Tp53* gene status in our *HIF1A* mice, but cDNA microarray analysis showed that some anti-apoptotic genes, which were reported as downstream targets of p53, were overexpressed in T cell lymphocytes obtained from *HIF1A* TG mice. Although a precise validation study is required, signals induced by HIF-1alpha overexpression may play an important role in lymphomagenesis in *HIF1A* TG mice in collaboration with anti-apoptotic pathway.

The role of HIF-1alpha in lymphomagenesis has recently been addressed. A c- Myc dependent B-cell lymphoma model showed that HIF-1alpha promoted tumor growth; loss of one *Hif1a* allele in *Tp53* deficient mice reduced the incidence of thymic lymphomas with delayed onset; and increased cell death was noted in *Hif1a* KO mice. Those data support our findings, and this *HIF1A* TG model will provide important information relative to occurrence and development of lymphoma.

Marzec et al reported that *NPM*/*ALK* chimeric gene, a causative gene abnormality in anaplastic large cell lymphoma (ALCL) in humans, induced up-regulation of HIF-1alpha in T cell lymphoma cells [32]. The lymphoma cells detected in our *HIF1A* TG mice showed T cell phenotype determined by FCM and clonal rearrangement of T cell receptors concomitant with an increase of angiogenesis in tumor tissues. In addition, we obtained preliminary results showing overexpression of HIF-1alpha in tumor tissues of human angioimmunoblastic T cell lymphoma (AITL). Since VEGF expression and a marked increase of small vessels in tumor tissue are common features in AITL, HIF-1alpha may play an important role in tumorigenesis of the lymphoma in humans.

## Supporting Information

Figure S1 **Hematopoietic potential of HIF1A TG mice.** (A) Serum concentration of erythropoietin in HIF1A TG or wild mice. (B) Complete blood counts of BALB/c, wild type, heterogygous or homogygous transgenic mice. (C) In vitro colony-forming assays were carried out in duplicate by plating 500 bone marrow cells with 1 ml of MethoCult™ M3434 medium, as described in Materials and Methods. Colonies were counted after 4–5 days.
(TIF)

Figure S2 **Fluorescence-activated cell sorter (FACS) analysis.** Lymphocytes from spleen (A), thymocytes, (B) obtained from 6-month-old mice were counted and stained with fluorochrome-conjugated monoclonal antibodies using standard procedures. Antibodies were anti-mouse against CD3e, CD4, CD8, CD19, B220, CD25, CD44, IgM (eBiosciences).
(PPTX)

Table S1 **Gene expression profile in lymphocytes obtained from TG mouse spleen.**
(DOCX)
