# Supplementary material for: Development of Lymphoproliferative Diseases by Hypoxia Inducible Factor-1alpha Is Associated with Prolonged Lymphocyte Survival
Source: PLoS One. 2013 Apr 12;8(4):e57833. doi: 10.1371/journal.pone.0057833 (PMC3625215; doi:10.1371/journal.pone.0057833)
Supplement: Table S1 — Gene expression profile in lymphocytes obtained from TG mouse spleen. (DOCX) [file pone.0057833.s003.docx]

**Table S1** Gene expression profile in lymphocytes obtained from TG mouse spleen.

Gene group Gene Fold expression

HIF-1alpha target genes EpoR 5.1

Myc 3.3

Brca2 11.9

Brca1 4.5

Apoptosis inhibitor 1 2.0

(bcl family) 2 7.7

3 2.1

4 7.0

5 5.8

6 2.7

7 2.0

Apoptosis inducer 8 0.8

9 0.9

DNA repair related 10 8.8

11 5.9

12 5.8

13 5.0

14 4.8

15 4.5

16 4.4

1600 genes >x2
